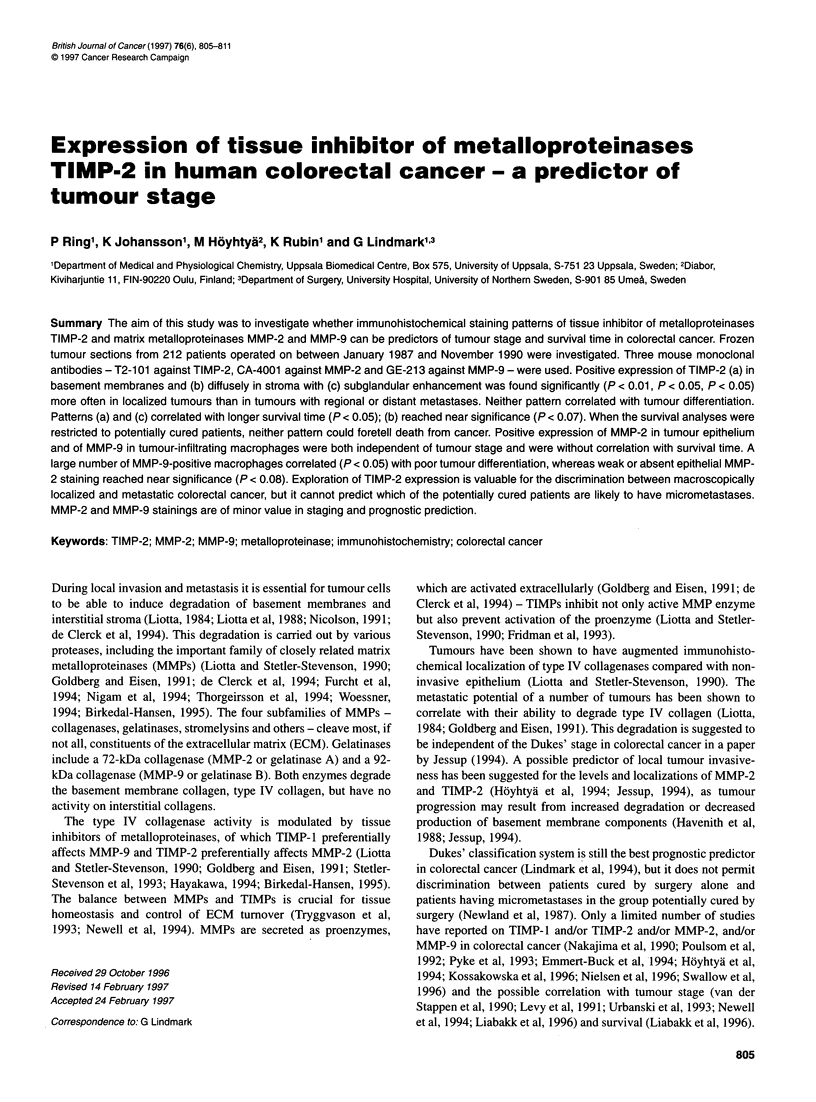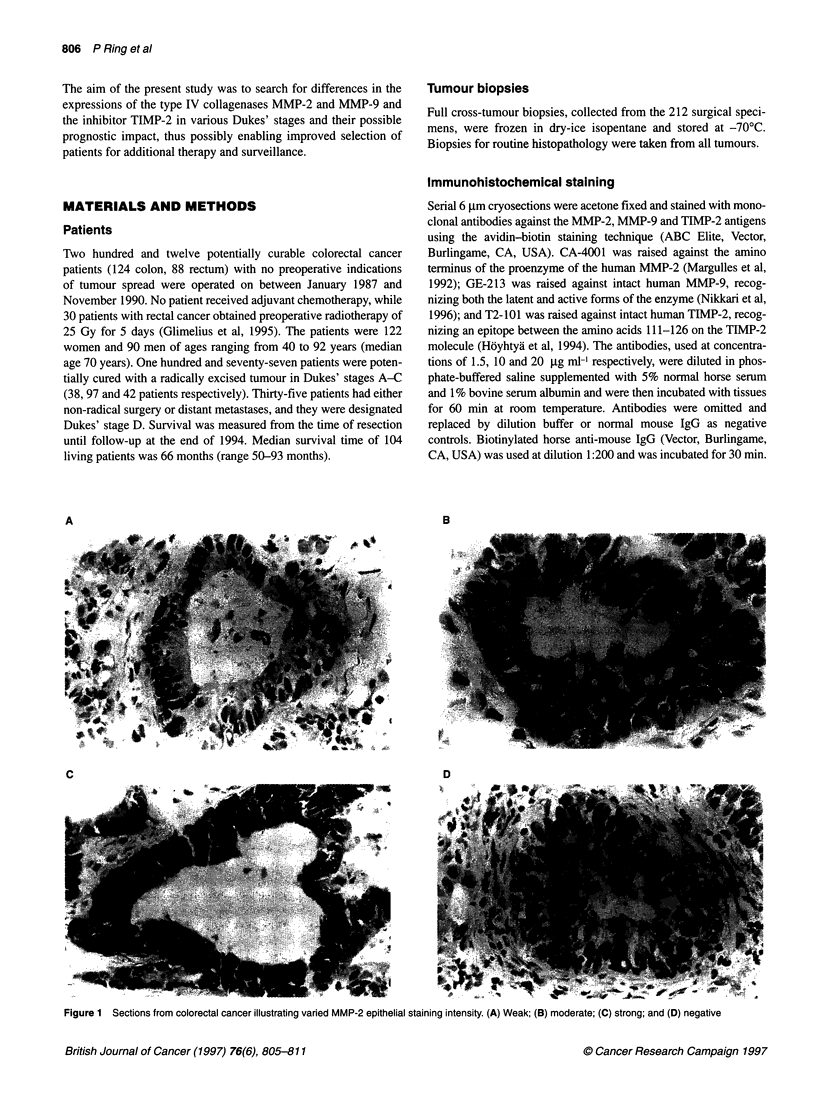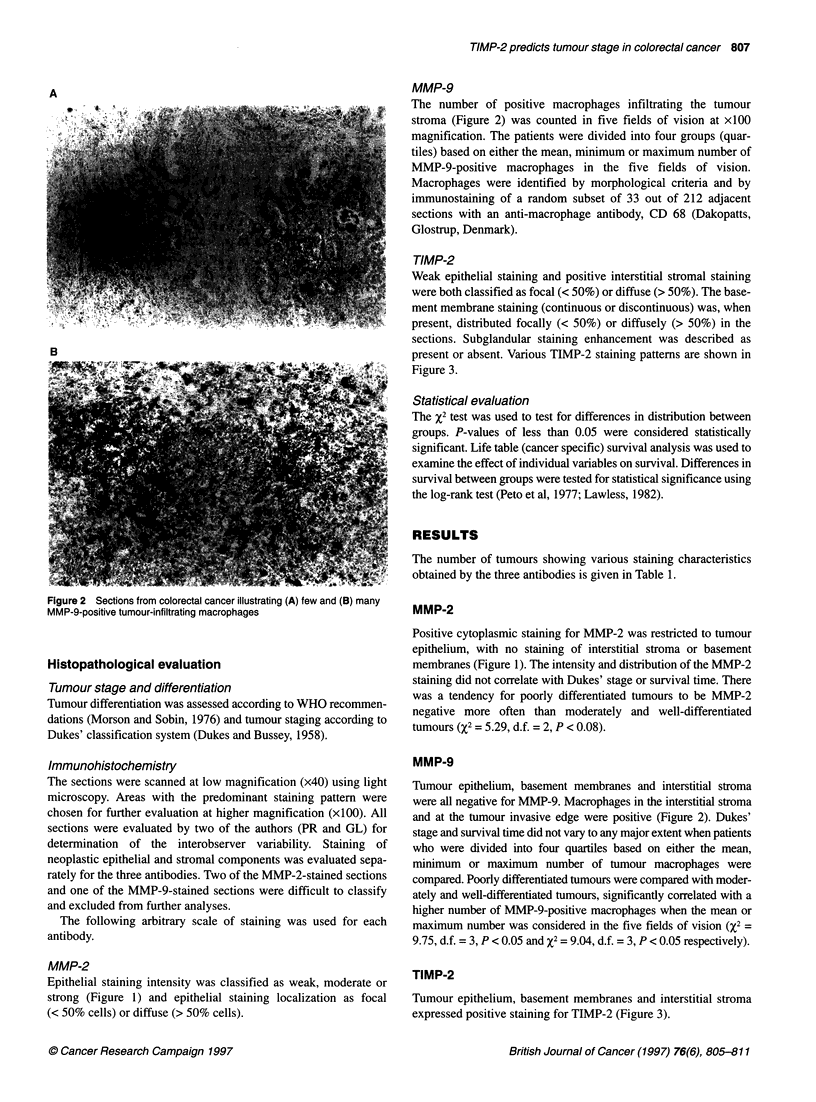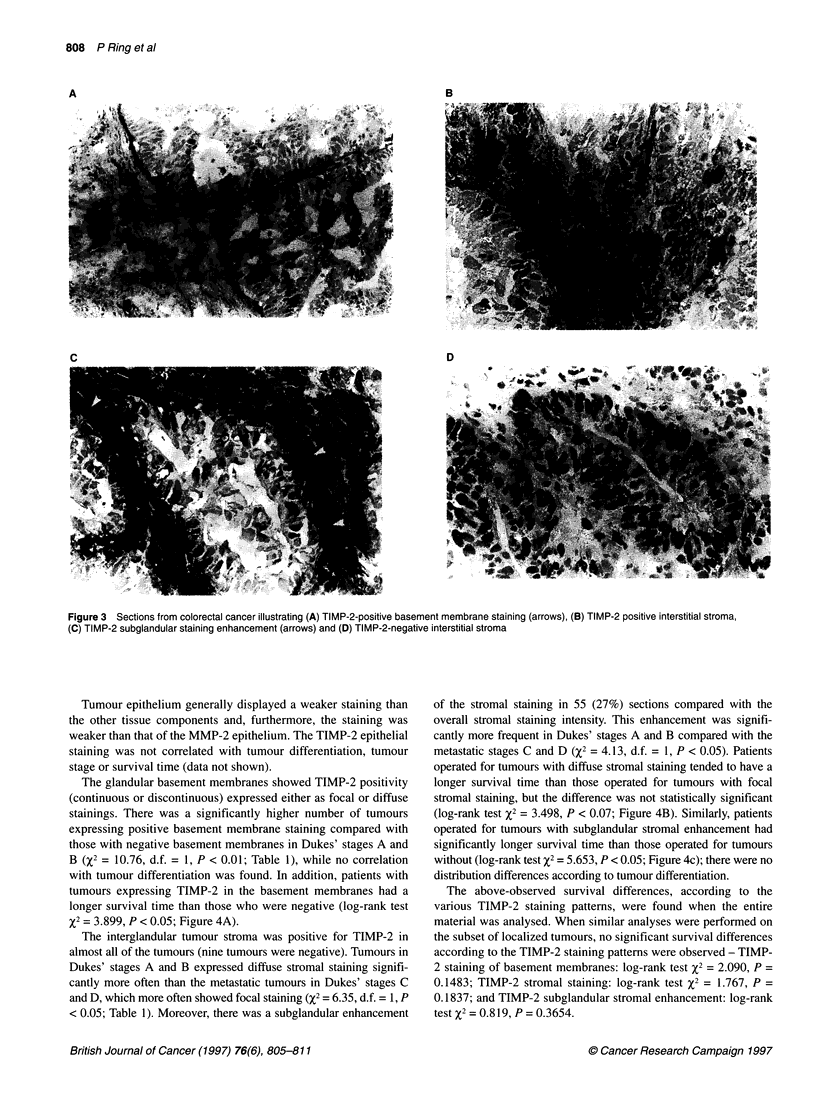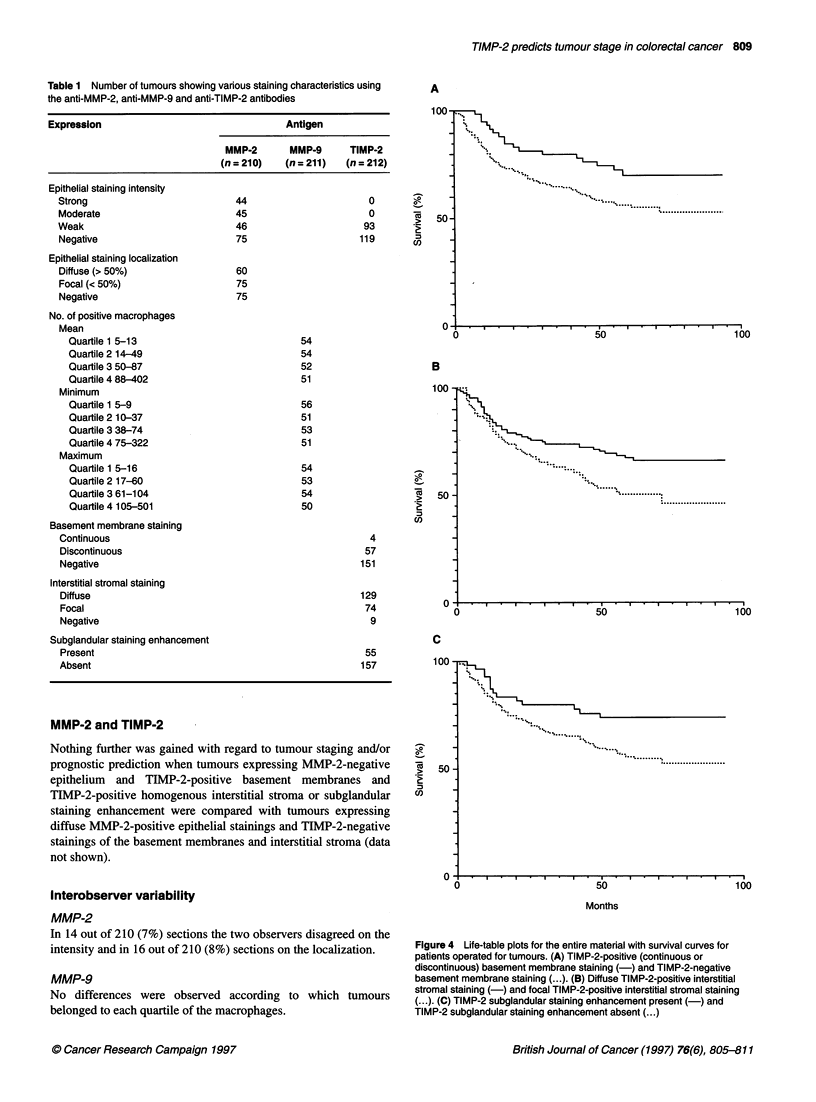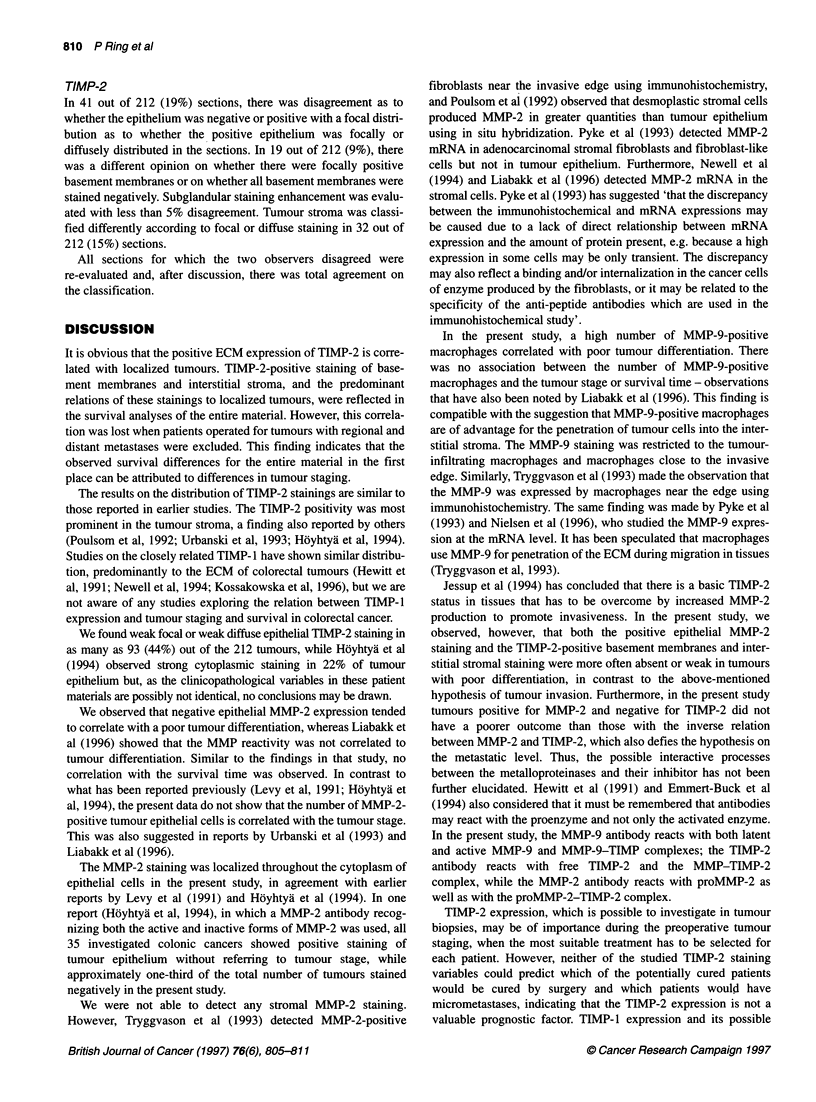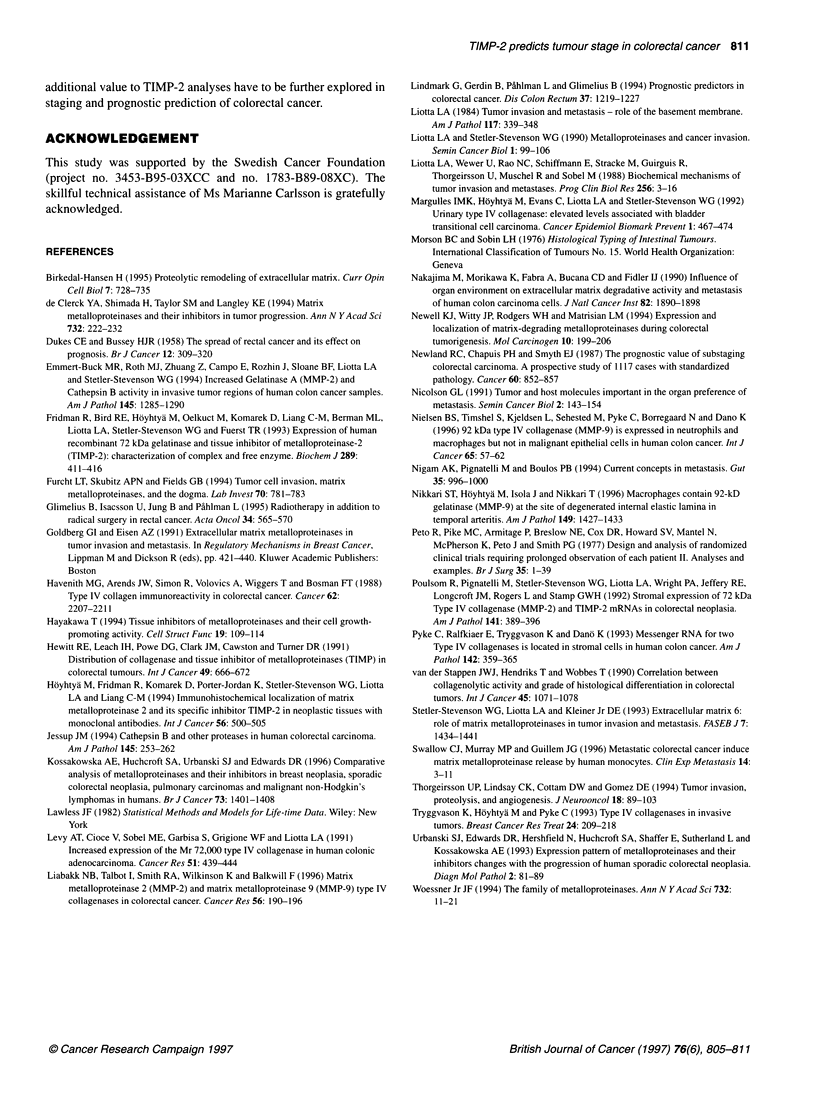# Expression of tissue inhibitor of metalloproteinases TIMP-2 in human colorectal cancer--a predictor of tumour stage.

**DOI:** 10.1038/bjc.1997.466

**Published:** 1997

**Authors:** P. Ring, K. Johansson, M. HÃ¶yhtyÃ¤, K. Rubin, G. Lindmark

**Affiliations:** Department of Medical and Physiological Chemistry, Uppsala Biomedical Centre, University of Uppsala, Sweden.

## Abstract

**Images:**


British Journal of Cancer (1997) 76(6), 805–811
© 1997 Cancer Research Campaign

### Expression of tissue inhibitor of metalloproteinases TIMP-2 in human colorectal cancer – a predictor of tumour stage

**P Ring[1], K Johansson[1], M Höyhtyä[2], K Rubin[1] and G Lindmark[1,3]**

[1]Department of Medical and Physiological Chemistry, Uppsala Biomedical Centre, Box 575, University of Uppsala, S-751 23 Uppsala, Sweden; [2]Diabor, Kiviharjuntie 11, FIN-90220 Oulu, Finland; [3]Department of Surgery, University Hospital, University of Northern Sweden, S-901 85 Umeå, Sweden

**Summary** The aim of this study was to investigate whether immunohistochemical staining patterns of tissue inhibitor of metalloproteinases TIMP-2 and matrix metalloproteinases MMP-2 and MMP-9 can be predictors of tumour stage and survival time in colorectal cancer. Frozen tumour sections from 212 patients operated on between January 1987 and November 1990 were investigated. Three mouse monoclonal antibodies – T2-101 against TIMP-2, CA-4001 against MMP-2 and GE-213 against MMP-9 – were used. Positive expression of TIMP-2 (a) in basement membranes and (b) diffusely in stroma with (c) subglandular enhancement was found significantly ($P < 0.01$, $P < 0.05$, $P < 0.05$) more often in localized tumours than in tumours with regional or distant metastases. Neither pattern correlated with tumour differentiation. Patterns (a) and (c) correlated with longer survival time ($P < 0.05$); (b) reached near significance ($P < 0.07$). When the survival analyses were restricted to potentially cured patients, neither pattern could foretell death from cancer. Positive expression of MMP-2 in tumour epithelium and of MMP-9 in tumour-infiltrating macrophages were both independent of tumour stage and were without correlation with survival time. A large number of MMP-9-positive macrophages correlated ($P < 0.05$) with poor tumour differentiation, whereas weak or absent epithelial MMP-2 staining reached near significance ($P < 0.08$). Exploration of TIMP-2 expression is valuable for the discrimination between macroscopically localized and metastatic colorectal cancer, but it cannot predict which of the potentially cured patients are likely to have micrometastases. MMP-2 and MMP-9 stainings are of minor value in staging and prognostic prediction.

**Keywords**: TIMP-2; MMP-2; MMP-9; metalloproteinase; immunohistochemistry; colorectal cancer

During local invasion and metastasis it is essential for tumour cells to be able to induce degradation of basement membranes and interstitial stroma (Liotta, 1984; Liotta et al, 1988; Nicolson, 1991; de Clerck et al, 1994). This degradation is carried out by various proteases, including the important family of closely related matrix metalloproteinases (MMPs) (Liotta and Stetler-Stevenson, 1990; Goldberg and Eisen, 1991; de Clerck et al, 1994; Furcht et al, 1994; Nigam et al, 1994; Thorgeirsson et al, 1994; Woessner, 1994; Birkedal-Hansen, 1995). The four subfamilies of MMPs – collagenases, gelatinases, stromelysins and others – cleave most, if not all, constituents of the extracellular matrix (ECM). Gelatinases include a 72-kDa collagenase (MMP-2 or gelatinase A) and a 92-kDa collagenase (MMP-9 or gelatinase B). Both enzymes degrade the basement membrane collagen, type IV collagen, but have no activity on interstitial collagens.

The type IV collagenase activity is modulated by tissue inhibitors of metalloproteinases, of which TIMP-1 preferentially affects MMP-9 and TIMP-2 preferentially affects MMP-2 (Liotta and Stetler-Stevenson, 1990; Goldberg and Eisen, 1991; Stetler-Stevenson et al, 1993; Hayakawa, 1994; Birkedal-Hansen, 1995). The balance between MMPs and TIMPs is crucial for tissue homeostasis and control of ECM turnover (Tryggvason et al, 1993; Newell et al, 1994). MMPs are secreted as proenzymes, which are activated extracellularly (Goldberg and Eisen, 1991; de Clerck et al, 1994) – TIMPs inhibit not only active MMP enzyme but also prevent activation of the proenzyme (Liotta and Stetler-Stevenson, 1990; Fridman et al, 1993).

Tumours have been shown to have augmented immunohistochemical localization of type IV collagenases compared with non-invasive epithelium (Liotta and Stetler-Stevenson, 1990). The metastatic potential of a number of tumours has been shown to correlate with their ability to degrade type IV collagen (Liotta, 1984; Goldberg and Eisen, 1991). This degradation is suggested to be independent of the Dukes' stage in colorectal cancer in a paper by Jessup (1994). A possible predictor of local tumour invasiveness has been suggested for the levels and localizations of MMP-2 and TIMP-2 (Höyhtyä et al, 1994; Jessup, 1994), as tumour progression may result from increased degradation or decreased production of basement membrane components (Havenith et al, 1988; Jessup, 1994).

Dukes' classification system is still the best prognostic predictor in colorectal cancer (Lindmark et al, 1994), but it does not permit discrimination between patients cured by surgery alone and patients having micrometastases in the group potentially cured by surgery (Newland et al, 1987). Only a limited number of studies have reported on TIMP-1 and/or TIMP-2 and/or MMP-2, and/or MMP-9 in colorectal cancer (Nakajima et al, 1990; Poulsom et al, 1992; Pyke et al, 1993; Emmert-Buck et al, 1994; Höyhtyä et al, 1994; Kossakowska et al, 1996; Nielsen et al, 1996; Swallow et al, 1996) and the possible correlation with tumour stage (van der Stappen et al, 1990; Levy et al, 1991; Urbanski et al, 1993; Newell et al, 1994; Liabakk et al, 1996) and survival (Liabakk et al, 1996).

*Received 29 October 1996*
*Revised 14 February 1997*
*Accepted 24 February 1997*

*Correspondence to:* G Lindmark

The aim of the present study was to search for differences in the expressions of the type IV collagenases MMP-2 and MMP-9 and the inhibitor TIMP-2 in various Dukes' stages and their possible prognostic impact, thus possibly enabling improved selection of patients for additional therapy and surveillance.

### MATERIALS AND METHODS

### Patients

Two hundred and twelve potentially curable colorectal cancer patients (124 colon, 88 rectum) with no preoperative indications of tumour spread were operated on between January 1987 and November 1990. No patient received adjuvant chemotherapy, while 30 patients with rectal cancer obtained preoperative radiotherapy of 25 Gy for 5 days (Glimelius et al, 1995). The patients were 122 women and 90 men of ages ranging from 40 to 92 years (median age 70 years). One hundred and seventy-seven patients were potentially cured with a radically excised tumour in Dukes' stages A–C (38, 97 and 42 patients respectively). Thirty-five patients had either non-radical surgery or distant metastases, and they were designated Dukes' stage D. Survival was measured from the time of resection until follow-up at the end of 1994. Median survival time of 104 living patients was 66 months (range 50–93 months).

### Tumour biopsies

Full cross-tumour biopsies, collected from the 212 surgical specimens, were frozen in dry-ice isopentane and stored at –70°C. Biopsies for routine histopathology were taken from all tumours.

### Immunohistochemical staining

Serial 6 μm cryosections were acetone fixed and stained with monoclonal antibodies against the MMP-2, MMP-9 and TIMP-2 antigens using the avidin–biotin staining technique (ABC Elite, Vector, Burlingame, CA, USA). CA-4001 was raised against the amino terminus of the proenzyme of the human MMP-2 (Margulles et al, 1992); GE-213 was raised against intact human MMP-9, recognizing both the latent and active forms of the enzyme (Nikkari et al, 1996); and T2-101 was raised against intact human TIMP-2, recognizing an epitope between the amino acids 111–126 on the TIMP-2 molecule (Höyhtyä et al, 1994). The antibodies, used at concentrations of 1.5, 10 and 20 μg $ml^{-1}$ respectively, were diluted in phosphate-buffered saline supplemented with 5% normal horse serum and 1% bovine serum albumin and were then incubated with tissues for 60 min at room temperature. Antibodies were omitted and replaced by dilution buffer or normal mouse IgG as negative controls. Biotinylated horse anti-mouse IgG (Vector, Burlingame, CA, USA) was used at dilution 1:200 and was incubated for 30 min.

**Figure 1** Sections from colorectal cancer illustrating varied MMP-2 epithelial staining intensity. (**A**) Weak; (**B**) moderate; (**C**) strong; and (**D**) negative

 © Cancer Research Campaign 1997

Figure 2 Sections from colorectal cancer illustrating (A) few and (B) many MMP-9-positive tumour-infiltrating macrophages

### Histopathological evaluation

*Tumour stage and differentiation*

Tumour differentiation was assessed according to WHO recommendations (Morson and Sobin, 1976) and tumour staging according to Dukes' classification system (Dukes and Bussey, 1958).

*Immunohistochemistry*

The sections were scanned at low magnification (×40) using light microscopy. Areas with the predominant staining pattern were chosen for further evaluation at higher magnification (×100). All sections were evaluated by two of the authors (PR and GL) for determination of the interobserver variability. Staining of neoplastic epithelial and stromal components was evaluated separately for the three antibodies. Two of the MMP-2-stained sections and one of the MMP-9-stained sections were difficult to classify and excluded from further analyses.

The following arbitrary scale of staining was used for each antibody.

*MMP-2*

Epithelial staining intensity was classified as weak, moderate or strong (Figure 1) and epithelial staining localization as focal (< 50% cells) or diffuse (> 50% cells).

*MMP-9*

The number of positive macrophages infiltrating the tumour stroma (Figure 2) was counted in five fields of vision at ×100 magnification. The patients were divided into four groups (quartiles) based on either the mean, minimum or maximum number of MMP-9-positive macrophages in the five fields of vision. Macrophages were identified by morphological criteria and by immunostaining of a random subset of 33 out of 212 adjacent sections with an anti-macrophage antibody, CD 68 (Dakopatts, Glostrup, Denmark).

*TIMP-2*

Weak epithelial staining and positive interstitial stromal staining were both classified as focal (< 50%) or diffuse (> 50%). The basement membrane staining (continuous or discontinuous) was, when present, distributed focally (< 50%) or diffusely (> 50%) in the sections. Subglandular staining enhancement was described as present or absent. Various TIMP-2 staining patterns are shown in Figure 3.

*Statistical evaluation*

The $\chi^2$ test was used to test for differences in distribution between groups. $P$-values of less than 0.05 were considered statistically significant. Life table (cancer specific) survival analysis was used to examine the effect of individual variables on survival. Differences in survival between groups were tested for statistical significance using the log-rank test (Peto et al, 1977; Lawless, 1982).

### RESULTS

The number of tumours showing various staining characteristics obtained by the three antibodies is given in Table 1.

### MMP-2

Positive cytoplasmic staining for MMP-2 was restricted to tumour epithelium, with no staining of interstitial stroma or basement membranes (Figure 1). The intensity and distribution of the MMP-2 staining did not correlate with Dukes' stage or survival time. There was a tendency for poorly differentiated tumours to be MMP-2 negative more often than moderately and well-differentiated tumours ($\chi^2 = 5.29$, d.f. = 2, $P < 0.08$).

### MMP-9

Tumour epithelium, basement membranes and interstitial stroma were all negative for MMP-9. Macrophages in the interstitial stroma and at the tumour invasive edge were positive (Figure 2). Dukes' stage and survival time did not vary to any major extent when patients who were divided into four quartiles based on either the mean, minimum or maximum number of tumour macrophages were compared. Poorly differentiated tumours were compared with moderately and well-differentiated tumours, significantly correlated with a higher number of MMP-9-positive macrophages when the mean or maximum number was considered in the five fields of vision ($\chi^2$ = 9.75, d.f. = 3, $P < 0.05$ and $\chi^2 = 9.04$, d.f. = 3, $P < 0.05$ respectively).

### TIMP-2

Tumour epithelium, basement membranes and interstitial stroma expressed positive staining for TIMP-2 (Figure 3).

© Cancer Research Campaign 1997

**Figure 3** Sections from colorectal cancer illustrating (**A**) TIMP-2-positive basement membrane staining (arrows), (**B**) TIMP-2 positive interstitial stroma, (**C**) TIMP-2 subglandular staining enhancement (arrows) and (**D**) TIMP-2-negative interstitial stroma

Tumour epithelium generally displayed a weaker staining than the other tissue components and, furthermore, the staining was weaker than that of the MMP-2 epithelium. The TIMP-2 epithelial staining was not correlated with tumour differentiation, tumour stage or survival time (data not shown).

The glandular basement membranes showed TIMP-2 positivity (continuous or discontinuous) expressed either as focal or diffuse stainings. There was a significantly higher number of tumours expressing positive basement membrane staining compared with those with negative basement membranes in Dukes' stages A and B ($\chi^2$ = 10.76, d.f. = 1, $P < 0.01$; Table 1), while no correlation with tumour differentiation was found. In addition, patients with tumours expressing TIMP-2 in the basement membranes had a longer survival time than those who were negative (log-rank test $\chi^2$ = 3.899, $P < 0.05$; Figure 4A).

The interglandular tumour stroma was positive for TIMP-2 in almost all of the tumours (nine tumours were negative). Tumours in Dukes' stages A and B expressed diffuse stromal staining significantly more often than the metastatic tumours in Dukes' stages C and D, which more often showed focal staining ($\chi^2$ = 6.35, d.f. = 1, $P < 0.05$; Table 1). Moreover, there was a subglandular enhancement of the stromal staining in 55 (27%) sections compared with the overall stromal staining intensity. This enhancement was significantly more frequent in Dukes' stages A and B compared with the metastatic stages C and D ($\chi^2$ = 4.13, d.f. = 1, $P < 0.05$). Patients operated for tumours with diffuse stromal staining tended to have a longer survival time than those operated for tumours with focal stromal staining, but the difference was not statistically significant (log-rank test $\chi^2$ = 3.498, $P < 0.07$; Figure 4B). Similarly, patients operated for tumours with subglandular stromal enhancement had significantly longer survival time than those operated for tumours without (log-rank test $\chi^2$ = 5.653, $P < 0.05$; Figure 4c); there were no distribution differences according to tumour differentiation.

The above-observed survival differences, according to the various TIMP-2 staining patterns, were found when the entire material was analysed. When similar analyses were performed on the subset of localized tumours, no significant survival differences according to the TIMP-2 staining patterns were observed – TIMP-2 staining of basement membranes: log-rank test $\chi^2$ = 2.090, $P$ = 0.1483; TIMP-2 stromal staining: log-rank test $\chi^2$ = 1.767, $P$ = 0.1837; and TIMP-2 subglandular stromal enhancement: log-rank test $\chi^2$ = 0.819, $P$ = 0.3654.

 © Cancer Research Campaign 1997

**Table 1** Number of tumours showing various staining characteristics using the anti-MMP-2, anti-MMP-9 and anti-TIMP-2 antibodies

| **Expression** | **Antigen** | | |
|---|---|---|---|
| | **MMP-2 (*n* = 210)** | **MMP-9 (*n* = 211)** | **TIMP-2 (*n* = 212)** |
| Epithelial staining intensity | | | |
| Strong | 44 | | 0 |
| Moderate | 45 | | 0 |
| Weak | 46 | | 93 |
| Negative | 75 | | 119 |
| Epithelial staining localization | | | |
| Diffuse (> 50%) | 60 | | |
| Focal (< 50%) | 75 | | |
| Negative | 75 | | |
| No. of positive macrophages | | | |
| Mean | | | |
| Quartile 1 5–13 | | 54 | |
| Quartile 2 14–49 | | 54 | |
| Quartile 3 50–87 | | 52 | |
| Quartile 4 88–402 | | 51 | |
| Minimum | | | |
| Quartile 1 5–9 | | 56 | |
| Quartile 2 10–37 | | 51 | |
| Quartile 3 38–74 | | 53 | |
| Quartile 4 75–322 | | 51 | |
| Maximum | | | |
| Quartile 1 5–16 | | 54 | |
| Quartile 2 17–60 | | 53 | |
| Quartile 3 61–104 | | 54 | |
| Quartile 4 105–501 | | 50 | |
| Basement membrane staining | | | |
| Continuous | | | 4 |
| Discontinuous | | | 57 |
| Negative | | | 151 |
| Interstitial stromal staining | | | |
| Diffuse | | | 129 |
| Focal | | | 74 |
| Negative | | | 9 |
| Subglandular staining enhancement | | | |
| Present | | | 55 |
| Absent | | | 157 |

### MMP-2 and TIMP-2

Nothing further was gained with regard to tumour staging and/or prognostic prediction when tumours expressing MMP-2-negative epithelium and TIMP-2-positive basement membranes and TIMP-2-positive homogenous interstitial stroma or subglandular staining enhancement were compared with tumours expressing diffuse MMP-2-positive epithelial stainings and TIMP-2-negative stainings of the basement membranes and interstitial stroma (data not shown).

### Interobserver variability

### *MMP-2*

In 14 out of 210 (7%) sections the two observers disagreed on the intensity and in 16 out of 210 (8%) sections on the localization.

### *MMP-9*

No differences were observed according to which tumours belonged to each quartile of the macrophages.

**Figure 4** Life-table plots for the entire material with survival curves for patients operated for tumours. (**A**) TIMP-2-positive (continuous or discontinuous) basement membrane staining (—) and TIMP-2-negative basement membrane staining (...). (**B**) Diffuse TIMP-2-positive interstitial stromal staining (—) and focal TIMP-2-positive interstitial stromal staining (...). (**C**) TIMP-2 subglandular staining enhancement present (—) and TIMP-2 subglandular staining enhancement absent (...)

*© Cancer Research Campaign 1997*

*TIMP-2*

In 41 out of 212 (19%) sections, there was disagreement as to whether the epithelium was negative or positive with a focal distribution as to whether the positive epithelium was focally or diffusely distributed in the sections. In 19 out of 212 (9%), there was a different opinion on whether there were focally positive basement membranes or on whether all basement membranes were stained negatively. Subglandular staining enhancement was evaluated with less than 5% disagreement. Tumour stroma was classified differently according to focal or diffuse staining in 32 out of 212 (15%) sections.

All sections for which the two observers disagreed were re-evaluated and, after discussion, there was total agreement on the classification.

### DISCUSSION

It is obvious that the positive ECM expression of TIMP-2 is correlated with localized tumours. TIMP-2-positive staining of basement membranes and interstitial stroma, and the predominant relations of these stainings to localized tumours, were reflected in the survival analyses of the entire material. However, this correlation was lost when patients operated for tumours with regional and distant metastases were excluded. This finding indicates that the observed survival differences for the entire material in the first place can be attributed to differences in tumour staging.

The results on the distribution of TIMP-2 stainings are similar to those reported in earlier studies. The TIMP-2 positivity was most prominent in the tumour stroma, a finding also reported by others (Poulsom et al, 1992; Urbanski et al, 1993; Höyhtyä et al, 1994). Studies on the closely related TIMP-1 have shown similar distribution, predominantly to the ECM of colorectal tumours (Hewitt et al, 1991; Newell et al, 1994; Kossakowska et al, 1996), but we are not aware of any studies exploring the relation between TIMP-1 expression and tumour staging and survival in colorectal cancer.

We found weak focal or weak diffuse epithelial TIMP-2 staining in as many as 93 (44%) out of the 212 tumours, while Höyhtyä et al (1994) observed strong cytoplasmic staining in 22% of tumour epithelium but, as the clinicopathological variables in these patient materials are possibly not identical, no conclusions may be drawn.

We observed that negative epithelial MMP-2 expression tended to correlate with a poor tumour differentiation, whereas Liabakk et al (1996) showed that the MMP reactivity was not correlated to tumour differentiation. Similar to the findings in that study, no correlation with the survival time was observed. In contrast to what has been reported previously (Levy et al, 1991; Höyhtyä et al, 1994), the present data do not show that the number of MMP-2-positive tumour epithelial cells is correlated with the tumour stage. This was also suggested in reports by Urbanski et al (1993) and Liabakk et al (1996).

The MMP-2 staining was localized throughout the cytoplasm of epithelial cells in the present study, in agreement with earlier reports by Levy et al (1991) and Höyhtyä et al (1994). In one report (Höyhtyä et al, 1994), in which a MMP-2 antibody recognizing both the active and inactive forms of MMP-2 was used, all 35 investigated colonic cancers showed positive staining of tumour epithelium without referring to tumour stage, while approximately one-third of the total number of tumours stained negatively in the present study.

We were not able to detect any stromal MMP-2 staining. However, Tryggvason et al (1993) detected MMP-2-positive fibroblasts near the invasive edge using immunohistochemistry, and Poulsom et al (1992) observed that desmoplastic stromal cells produced MMP-2 in greater quantities than tumour epithelium using in situ hybridization. Pyke et al (1993) detected MMP-2 mRNA in adenocarcinomal stromal fibroblasts and fibroblast-like cells but not in tumour epithelium. Furthermore, Newell et al (1994) and Liabakk et al (1996) detected MMP-2 mRNA in the stromal cells. Pyke et al (1993) has suggested 'that the discrepancy between the immunohistochemical and mRNA expressions may be caused due to a lack of direct relationship between mRNA expression and the amount of protein present, e.g. because a high expression in some cells may be only transient. The discrepancy may also reflect a binding and/or internalization in the cancer cells of enzyme produced by the fibroblasts, or it may be related to the specificity of the anti-peptide antibodies which are used in the immunohistochemical study'.

In the present study, a high number of MMP-9-positive macrophages correlated with poor tumour differentiation. There was no association between the number of MMP-9-positive macrophages and the tumour stage or survival time – observations that have also been noted by Liabakk et al (1996). This finding is compatible with the suggestion that MMP-9-positive macrophages are of advantage for the penetration of tumour cells into the interstitial stroma. The MMP-9 staining was restricted to the tumour-infiltrating macrophages and macrophages close to the invasive edge. Similarly, Tryggvason et al (1993) made the observation that the MMP-9 was expressed by macrophages near the edge using immunohistochemistry. The same finding was made by Pyke et al (1993) and Nielsen et al (1996), who studied the MMP-9 expression at the mRNA level. It has been speculated that macrophages use MMP-9 for penetration of the ECM during migration in tissues (Tryggvason et al, 1993).

Jessup et al (1994) has concluded that there is a basic TIMP-2 status in tissues that has to be overcome by increased MMP-2 production to promote invasiveness. In the present study, we observed, however, that both the positive epithelial MMP-2 staining and the TIMP-2-positive basement membranes and interstitial stromal staining were more often absent or weak in tumours with poor differentiation, in contrast to the above-mentioned hypothesis of tumour invasion. Furthermore, in the present study tumours positive for MMP-2 and negative for TIMP-2 did not have a poorer outcome than those with the inverse relation between MMP-2 and TIMP-2, which also defies the hypothesis on the metastatic level. Thus, the possible interactive processes between the metalloproteinases and their inhibitor has not been further elucidated. Hewitt et al (1991) and Emmert-Buck et al (1994) also considered that it must be remembered that antibodies may react with the proenzyme and not only the activated enzyme. In the present study, the MMP-9 antibody reacts with both latent and active MMP-9 and MMP-9–TIMP complexes; the TIMP-2 antibody reacts with free TIMP-2 and the MMP–TIMP-2 complex, while the MMP-2 antibody reacts with proMMP-2 as well as with the proMMP-2–TIMP-2 complex.

TIMP-2 expression, which is possible to investigate in tumour biopsies, may be of importance during the preoperative tumour staging, when the most suitable treatment has to be selected for each patient. However, neither of the studied TIMP-2 staining variables could predict which of the potentially cured patients would be cured by surgery and which patients would have micrometastases, indicating that the TIMP-2 expression is not a valuable prognostic factor. TIMP-1 expression and its possible

© Cancer Research Campaign 1997

additional value to TIMP-2 analyses have to be further explored in staging and prognostic prediction of colorectal cancer.

### ACKNOWLEDGEMENT

This study was supported by the Swedish Cancer Foundation (project no. 3453-B95-03XCC and no. 1783-B89-08XC). The skillful technical assistance of Ms Marianne Carlsson is gratefully acknowledged.

### REFERENCES

Birkedal-Hansen H (1995) Proteolytic remodeling of extracellular matrix. *Curr Opin Cell Biol* **7**: 728–735

de Clerck YA, Shimada H, Taylor SM and Langley KE (1994) Matrix metalloproteinases and their inhibitors in tumor progression. *Ann N Y Acad Sci* **732**: 222–232

Dukes CE and Bussey HJR (1958) The spread of rectal cancer and its effect on prognosis. *Br J Cancer* **12**: 309–320

Emmert-Buck MR, Roth MJ, Zhuang Z, Campo E, Rozhin J, Sloane BF, Liotta LA and Stetler-Stevenson WG (1994) Increased Gelatinase A (MMP-2) and Cathepsin B activity in invasive tumor regions of human colon cancer samples. *Am J Pathol* **145**: 1285–1290

Fridman R, Bird RE, Höyhtyä M, Oelkuct M, Komarek D, Liang C-M, Berman ML, Liotta LA, Stetler-Stevenson WG and Fuerst TR (1993) Expression of human recombinant 72 kDa gelatinase and tissue inhibitor of metalloproteinase-2 (TIMP-2): characterization of complex and free enzyme. *Biochem J* **289**: 411–416

Furcht LT, Skubitz APN and Fields GB (1994) Tumor cell invasion, matrix metalloproteinases, and the dogma. *Lab Invest* **70**: 781–783

Glimelius B, Isacsson U, Jung B and Påhlman L (1995) Radiotherapy in addition to radical surgery in rectal cancer. *Acta Oncol* **34**: 565–570

Goldberg GI and Eisen AZ (1991) Extracellular matrix metalloproteinases in tumor invasion and metastasis. In *Regulatory Mechanisms in Breast Cancer*, Lippman M and Dickson R (eds), pp. 421–440. Kluwer Academic Publishers: Boston

Havenith MG, Arends JW, Simon R, Volovics A, Wiggers T and Bosman FT (1988) Type IV collagen immunoreactivity in colorectal cancer. *Cancer* **62**: 2207–2211

Hayakawa T (1994) Tissue inhibitors of metalloproteinases and their cell growth-promoting activity. *Cell Struct Func* **19**: 109–114

Hewitt RE, Leach IH, Powe DG, Clark JM, Cawston and Turner DR (1991) Distribution of collagenase and tissue inhibitor of metalloproteinases (TIMP) in colorectal tumours. *Int J Cancer* **49**: 666–672

Höyhtyä M, Fridman R, Komarek D, Porter-Jordan K, Stetler-Stevenson WG, Liotta LA and Liang C-M (1994) Immunohistochemical localization of matrix metalloproteinase 2 and its specific inhibitor TIMP-2 in neoplastic tissues with monoclonal antibodies. *Int J Cancer* **56**: 500–505

Jessup JM (1994) Cathepsin B and other proteases in human colorectal carcinoma. *Am J Pathol* **145**: 253–262

Kossakowska AE, Huchcroft SA, Urbanski SJ and Edwards DR (1996) Comparative analysis of metalloproteinases and their inhibitors in breast neoplasia, sporadic colorectal neoplasia, pulmonary carcinomas and malignant non-Hodgkin's lymphomas in humans. *Br J Cancer* **73**: 1401–1408

Lawless JF (1982) *Statistical Methods and Models for Life-time Data*. Wiley: New York

Levy AT, Cioce V, Sobel ME, Garbisa S, Grigione WF and Liotta LA (1991) Increased expression of the Mr 72,000 type IV collagenase in human colonic adenocarcinoma. *Cancer Res* **51**: 439–444

Liabakk NB, Talbot I, Smith RA, Wilkinson K and Balkwill F (1996) Matrix metalloproteinase 2 (MMP-2) and matrix metalloproteinase 9 (MMP-9) type IV collagenases in colorectal cancer. *Cancer Res* **56**: 190–196

Lindmark G, Gerdin B, Påhlman L and Glimelius B (1994) Prognostic predictors in colorectal cancer. *Dis Colon Rectum* **37**: 1219–1227

Liotta LA (1984) Tumor invasion and metastasis – role of the basement membrane. *Am J Pathol* **117**: 339–348

Liotta LA and Stetler-Stevenson WG (1990) Metalloproteinases and cancer invasion. *Semin Cancer Biol* **1**: 99–106

Liotta LA, Wewer U, Rao NC, Schiffmann E, Stracke M, Guirguis R, Thorgeirsson U, Muschel R and Sobel M (1988) Biochemical mechanisms of tumor invasion and metastases. *Prog Clin Biol Res* **256**: 3–16

Margulles IMK, Höyhtyä M, Evans C, Liotta LA and Stetler-Stevenson WG (1992) Urinary type IV collagenase: elevated levels associated with bladder transitional cell carcinoma. *Cancer Epidemiol Biomark Prevent* **1**: 467–474

Morson BC and Sobin LH (1976) *Histological Typing of Intestinal Tumours*. International Classification of Tumours No. 15. World Health Organization: Geneva

Nakajima M, Morikawa K, Fabra A, Bucana CD and Fidler IJ (1990) Influence of organ environment on extracellular matrix degradative activity and metastasis of human colon carcinoma cells. *J Natl Cancer Inst* **82**: 1890–1898

Newell KJ, Witty JP, Rodgers WH and Matrisian LM (1994) Expression and localization of matrix-degrading metalloproteinases during colorectal tumorigenesis. *Mol Carcinogen* **10**: 199–206

Newland RC, Chapuis PH and Smyth EJ (1987) The prognostic value of substaging colorectal carcinoma. A prospective study of 1117 cases with standardized pathology. *Cancer* **60**: 852–857

Nicolson GL (1991) Tumor and host molecules important in the organ preference of metastasis. *Semin Cancer Biol* **2**: 143–154

Nielsen BS, Timshel S, Kjeldsen L, Sehested M, Pyke C, Borregaard N and Dano K (1996) 92 kDa type IV collagenase (MMP-9) is expressed in neutrophils and macrophages but not in malignant epithelial cells in human colon cancer. *Int J Cancer* **65**: 57–62

Nigam AK, Pignatelli M and Boulos PB (1994) Current concepts in metastasis. *Gut* **35**: 996–1000

Nikkari ST, Höyhtyä M, Isola J and Nikkari T (1996) Macrophages contain 92-kD gelatinase (MMP-9) at the site of degenerated internal elastic lamina in temporal arteritis. *Am J Pathol* **149**: 1427–1433

Peto R, Pike MC, Armitage P, Breslow NE, Cox DR, Howard SV, Mantel N, McPherson K, Peto J and Smith PG (1977) Design and analysis of randomized clinical trials requiring prolonged observation of each patient II. Analyses and examples. *Br J Surg* **35**: 1–39

Poulsom R, Pignatelli M, Stetler-Stevenson WG, Liotta LA, Wright PA, Jeffery RE, Longcroft JM, Rogers L and Stamp GWH (1992) Stromal expression of 72 kDa Type IV collagenase (MMP-2) and TIMP-2 mRNAs in colorectal neoplasia. *Am J Pathol* **141**: 389–396

Pyke C, Ralfkiaer E, Tryggvason K and Danö K (1993) Messenger RNA for two Type IV collagenases is located in stromal cells in human colon cancer. *Am J Pathol* **142**: 359–365

van der Stappen JWJ, Hendriks T and Wobbes T (1990) Correlation between collagenolytic activity and grade of histological differentiation in colorectal tumors. *Int J Cancer* **45**: 1071–1078

Stetler-Stevenson WG, Liotta LA and Kleiner Jr DE (1993) Extracellular matrix 6: role of matrix metalloproteinases in tumor invasion and metastasis. *FASEB J* **7**: 1434–1441

Swallow CJ, Murray MP and Guillem JG (1996) Metastatic colorectal cancer induce matrix metalloproteinase release by human monocytes. *Clin Exp Metastasis* **14**: 3–11

Thorgeirsson UP, Lindsay CK, Cottam DW and Gomez DE (1994) Tumor invasion, proteolysis, and angiogenesis. *J Neurooncol* **18**: 89–103

Tryggvason K, Höyhtyä M and Pyke C (1993) Type IV collagenases in invasive tumors. *Breast Cancer Res Treat* **24**: 209–218

Urbanski SJ, Edwards DR, Hershfield N, Huchcroft SA, Shaffer E, Sutherland L and Kossakowska AE (1993) Expression pattern of metalloproteinases and their inhibitors changes with the progression of human sporadic colorectal neoplasia. *Diagn Mol Pathol* **2**: 81–89

Woessner Jr JF (1994) The family of metalloproteinases. *Ann N Y Acad Sci* **732**: 11–21

© Cancer Research Campaign 1997